# A Microwell-Based Intestinal Organoid-Macrophage Co-Culture System to Study Intestinal Inflammation

**DOI:** 10.3390/ijms232315364

**Published:** 2022-12-06

**Authors:** Panagiota Kakni, Roman Truckenmüller, Pamela Habibović, Martijn van Griensven, Stefan Giselbrecht

**Affiliations:** 1 Department of Instructive Biomaterials Engineering, MERLN Institute for Technology-Inspired Regenerative Medicine, Maastricht University, Universiteitssingel 40, 6229 ER Maastricht, The Netherlands; 2 Department of Cell Biology-Inspired Tissue Engineering, MERLN Institute for Technology-Inspired Regenerative Medicine, Maastricht University, Universiteitssingel 40, 6229 ER Maastricht, The Netherlands

**Keywords:** intestinal organoids, immune system, microwells, macrophages, co-culture

## Abstract

The mammalian intestinal epithelium contains more immune cells than any other tissue, and this is largely because of its constant exposure to pathogens. Macrophages are crucial for maintaining intestinal homeostasis, but they also play a central role in chronic pathologies of the digestive system. We developed a versatile microwell-based intestinal organoid-macrophage co-culture system that enables us to recapitulate features of intestinal inflammation. This microwell-based platform facilitates the controlled positioning of cells in different configurations, continuous in situ monitoring of cell interactions, and high-throughput downstream applications. Using this novel system, we compared the inflammatory response when intestinal organoids were co-cultured with macrophages versus when intestinal organoids were treated with the pro-inflammatory cytokine TNF-α. Furthermore, we demonstrated that the tissue-specific response differs according to the physical distance between the organoids and the macrophages and that the intestinal organoids show an immunomodulatory competence. Our novel microwell-based intestinal organoid model incorporating acellular and cellular components of the immune system can pave the way to unravel unknown mechanisms related to intestinal homeostasis and disorders.

## 1. Introduction

The intestine comprises the largest compartment of the immune system, due to its continuous exposure to foreign antigens [1]. Immunological processes mainly take place in the mucosa that consists of the epithelium, the lamina propria, and the muscularis mucosa. The crosstalk between immune cells and intestinal epithelial cells is important for gut homeostasis and alterations can result in inflammatory diseases, such as inflammatory bowel disease (IBD), which is a group of chronic inflammatory diseases of the digestive tract. This term is used to describe both ulcerative colitis and Crohn’s disease, among others [2]. The pathogenesis of IBD involves a complex interplay among genetic, epigenetic, immunological and microbiological factors, and epithelial barrier dysfunction [2,3]. A lot of interest has been shifted toward the role of the immune system in the pathogenesis of IBD. In this disease, increased infiltration of inflammatory cells into the lamina propria and submucosa of the intestine is observed, accompanied by an increased expression of pro-inflammatory cytokines, such as interleukin-1 beta (IL-1β) and tumor necrosis factor alpha (TNF-α). These observations have led to the development of clinical immunomodulatory therapies, such as the treatment with infliximab. This is a monoclonal antibody that binds with high affinity to TNF-α and neutralizes its biological activity, thus reducing inflammation [4,5]. However, patients often do not respond to such treatments. Thus, it is imperative to gain a better insight into the involvement of epithelial and other factors related to the pathogenesis of IBD [3]. Although mouse models have provided invaluable information over the years, they mostly focus on one aspect of IBD and they cannot fully recapitulate the complexity of human diseases [6,7]. Hence, establishing in vitro models that mimic both the epithelial and immune compartments is necessary to develop more effective therapies [8].

Intestinal organoids are self-organizing, three-dimensional (3D) mini-organs that can be derived from stem cells and recapitulate multiple features of the in vivo intestine [9,10]. Specifically, they have a multicellular composition, they are organized into crypt-villus structures, and they are able to perform intestine-specific functions such as barrier formation and nutrient uptake [11]. Organoids recapitulate intestinal properties much closer than 2D monolayer systems (e.g., using Caco-2 cells) and allow for in-depth analysis of pathogen–host interactions and investigation of mechanisms related to development and disease. However, organoid systems usually lack immune system components; thus, their applicability for investigating the mechanisms underlying certain diseases and disease modeling is limited [12].

In this study, we aimed to establish an intestinal organoid model to study the interactions of the epithelium with immune cells and to recapitulate aspects of intestinal inflammation. Hence, we developed a microwell-based co-culture system of mouse intestinal organoids and RAW 264.7 macrophages. This microwell-based co-culture system is highly versatile and facilitates the controlled positioning of different cell types in multiple configurations, the continuous monitoring of cell interactions during co-culture, and high-throughput downstream applications. Additionally, there is no viscous and ill-defined hydrogel matrix (e.g., basement membrane extract) that could interfere with or slow down the contact or interactions between the cells. Intestinal macrophages play a crucial role in intestinal immunity and homeostasis, but also in the development of intestinal inflammation [13,14]. When pathogens invade the intestinal epithelium, immune cells, mainly macrophages, become activated and they release a series of pro-inflammatory cytokines, such as TNF-α, IL1, and IL6 [14]. Cytokines can affect the epithelium both positively and negatively. They can induce or restrict cell proliferation or cell death and also alter the barrier permeability [15]. For example, cytokines mediate mucosal healing by controlling the epithelial cell activation, differentiation, survival, and migration [16]. However, aberrant and excessive secretion of those factors leads to chronic inflammation, a typical feature of IBD.

Using our microwell-based organoid culture model [17], we established a direct and an indirect co-culture system of mouse intestinal organoids with RAW 264.7 cells and compared the effects with TNF-α treatment, which is known to be the first cytokine secreted in the inflammation cascade [18]. We studied the effects of different numbers of macrophages on the intestinal organoids and compared them with organoids treated with different concentrations of TNF-α. We also showed that there are prominent differences when placing the macrophages in close proximity to the organoids, by comparing the direct (juxtacrine and paracrine signaling) with the indirect co-culture system (paracrine signaling). A quantification of a subpanel of cytokines, which are likely involved in the interactions between the intestinal epithelium and macrophages, was performed to identify other key players next to TNF-α. These experiments also revealed that organoids have an innate immunomodulatory function upon exposure to inflammatory cytokines.

## 2. Results

### 2.1. Monocultures of Intestinal Organoids and RAW 264.7 Macrophages in Microwell Arrays

**Figure 1:**
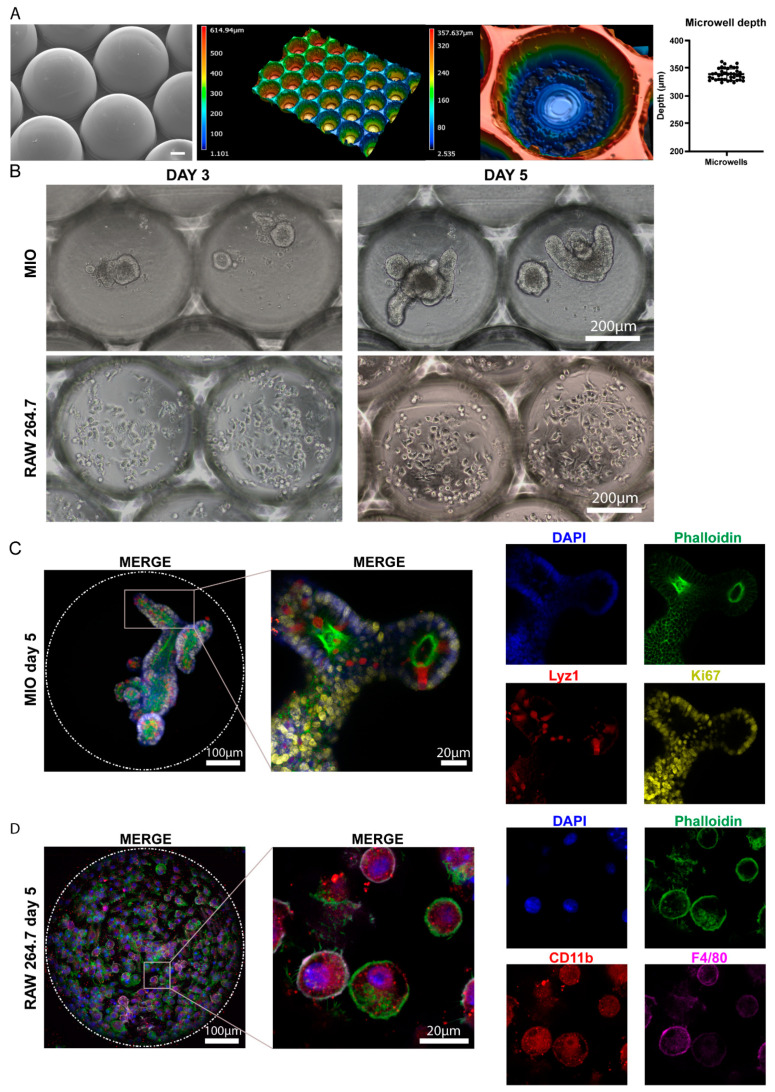
Monocultures of organoids (MIO) and RAW 264.7 cells in microwells. (**A**) Characterization of the thermoformed microwell arrays was performed using SEM (Jeol, Nieuw-Vennep, Benelux) and confocal laser scanning microscopy (Leica, Wetzlar, Germany). The graph indicates the depth of the microwells. Data are presented as the mean  ±  SEM. (*n* = 4). Scale bar represents 100 μm. (**B**) Bright-field images demonstrating the growth of the organoids (top) and macrophages (bottom) over a period of 5 days. (**C**) Immunofluorescence stainings of intestinal organoids grown in microwells for the F-actin marker phalloidin (green), the Paneth cell marker Lyz1 (red), and the proliferation marker Ki67 (yellow). Dashed circle represents the circumference of the microwells. The white square represents the area magnified in the respective column to the right. (**D**) Immunofluorescence stainings of RAW 264.7 cells grown in microwells for phalloidin (green) and the macrophage markers CD11b (red) and F4/80 (magenta). Dashed circle represents the circumference of the microwells. The white square represents the area magnified in the respective column to the right. Abbreviations: MIO, mouse intestinal organoid; DAPI, 4′,6-diamidino-2-phenylindole.

Following microthermoforming, we performed characterization of the microwells. We verified that each microwell had a diameter of 500 μm and a depth of approximately 300 μm (Figure 1A). Our group has previously shown that these dimensions are appropriate for the culture of intestinal organoids [17]. Thus, we decided to use microwells with the same size to further advance this microwell-based organoid model by incorporating immune cells.

Prior to co-culture experiments, we assessed the efficiency of monocultures of organoids and macrophages. Our group has previously established a method to culture intestinal organoids in polymer-film-based microwell arrays [17]. Here, we further improved this method by reducing the amount of Matrigel supplemented in the medium from 5% to 2%. This can be beneficial for the sequential seeding of different cells, where a medium with lower viscosity is superior to a medium with higher viscosity. After 5 days in culture, organoids demonstrated a crypt-villus architecture (Figure 1B), which is consistent with the architecture of organoids embedded in Matrigel [9] and the ones grown in microwells with 5% Matrigel [17]. Immunofluorescence stainings indicated that organoids maintained intestinal phenotypic characteristics as well (Figure 1C). Specifically, the expression of phalloidin, which marks the F-actin located at the apical side of the epithelium, was identified at the inner surface of the organoids facing the organoid lumen [17]. Paneth cells, marked by lysozyme, were found adjacent to the proliferating cells (marked by Ki67) as suggested by their protective role [19]. Collectively, these results indicate that intestinal organoids can be successfully cultured in microwell arrays with reduced amounts of Matrigel.

Following that, we cultured RAW 264.7 cells (50,000 cells/well) in microwells using IntestiCult medium (STEMCELL Technologies, Vancouver, BC, Canada). This was performed to evaluate whether these macrophages survive in the organoid medium and maintain their phenotype. Bright-field images showed that RAW 264.7 macrophages attached to the inner surface of the microwells and maintained their normal cell morphology (Figure 1B). Immunofluorescence stainings demonstrated that these cells expressed the typical macrophage markers CD11b and F4/80, thus indicating that they maintain the macrophage phenotype (Figure 1D). Overall, these data confirm that RAW 264.7 cells can be efficiently cultured in microwell arrays, even in organoid medium.

### 2.2. Direct Co-Culture of Intestinal Organoids and RAW 264.7 Macrophages in Comparison with TNF-α Treatment of Intestinal Organoid Monocultures

**Figure 2:**
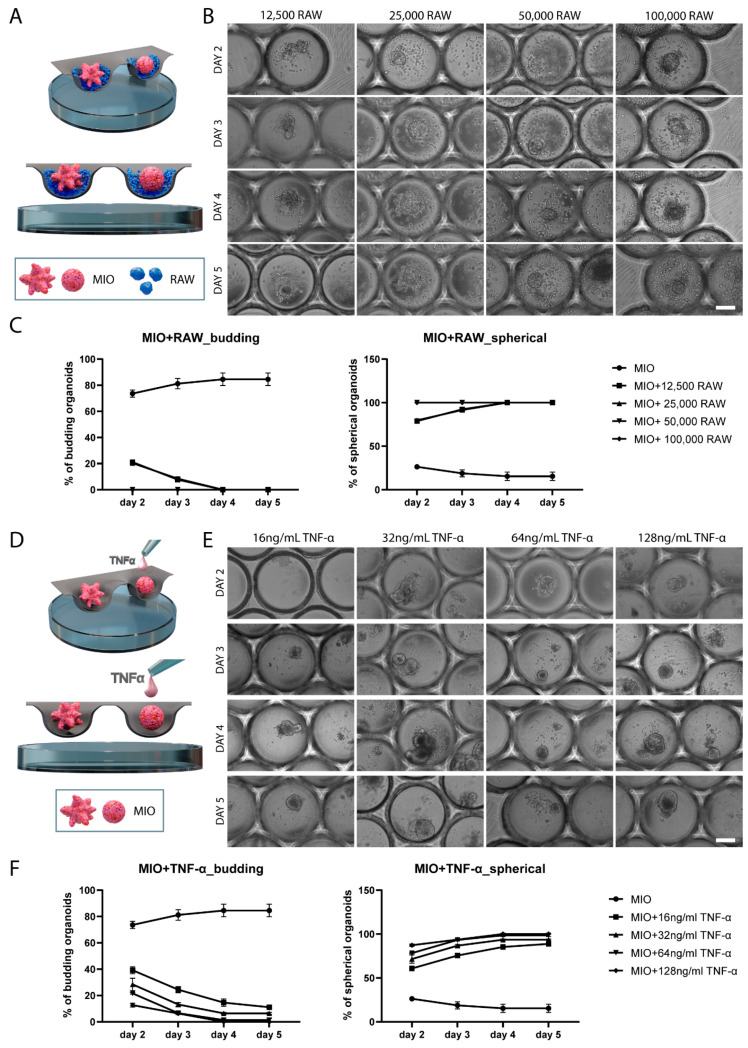
Morphological changes in organoids upon co-culture with macrophages or treatment with TNF-α. (**A**) Schematic illustration of the direct co-culture of intestinal organoids with RAW 264.7 cells in microwell arrays. (**B**) Representative bright-field images demonstrating the morphological changes in organoids upon co-culture with different amounts of macrophages (12,500, 25,000, 50,000, and 100,000 cells) over a period of 5 days. Scale bar represents 200 μm. (**C**) Percentages of crypt-villus-structured and spherical organoids upon direct co-culture. Data are presented as the mean  ±  SEM (*n* = 3). (**D**) Schematic illustration of intestinal organoids treated with TNF-α. (**E**) Representative bright-field images demonstrating the morphological changes in organoids upon treatment with different concentrations of TNF-α (16, 32, 64, and 128 ng/mL) over a period of 5 days. Scale bar represents 200 μm. (**F**) Percentages of budded and spherical organoids upon TNF-α treatment. Data presented as mean ± S.E.M. (*n* = 3). Abbreviations: MIO, mouse intestinal organoids; RAW, RAW 264.7 cells; TNF-α, tumor necrosis factor alpha.

The intestine in vivo is constantly exposed to foreign antigens; hence, it accommodates the largest compartment of the immune system [20]. Macrophages play a crucial role in the maintenance of intestinal homeostasis and they provide a first line of innate immune defense. They are located under the epithelial monolayer, an ideal position to identify and eradicate any pathogen that crossed the epithelium [20]. When a pathogenic event occurs, apart from the tissue resident macrophages, additional macrophages infiltrate the intestinal mucosa and release cytokines [14]. However, when excessive secretion of cytokines persists, it leads to chronic inflammation, which is a characteristic of IBD. To create an in vitro model that mimics features of the intestinal inflammation, we developed here a 3D co-culture system in microwell arrays, in which mouse intestinal organoids are co-cultured in very close proximity and even in direct contact with the murine macrophage cell line RAW 264.7 (Figure 2A, Supplementary Figure S1 and Supplementary video S1). In this co-culture system, there are both juxtacrine and paracrine cell signaling and it will be referred to as ‘direct co-culture’ (Supplementary Table S1).

Upon direct co-culture of organoids with different amounts of macrophages, we observed changes in the organoids’ morphology (Figure 2B). Specifically, organoids gradually lost their crypt-villus architecture and turned to spherical structures. Bright-field imaging with subsequent quantification showed that increasing numbers of RAW 264.7 cells had a faster and more prominent effect on the change in the organoid morphology from a crypt-villus to a more spherical shape (Figure 2C).

One of the mechanisms mediating these morphological changes is the release of TNF-α from the macrophages [21,22,23]. TNF-α is a pro-inflammatory cytokine that affects the intestinal epithelial permeability by loosening the junctions between cells and, hence, it has been associated with diarrhea during inflammation [24,25,26]. To investigate further whether the change in organoid morphology is solely induced by TNF-α, we treated the organoids in a monoculture with TNF-α and evaluated their morphology over a period of 5 days (Figure 2D). Different concentrations of TNF-α were added to the organoid medium, ranging from 16 ng/mL to 128 ng/mL (Figure 2E). According to Hahn et al. 2017, treatment of organoids with less than 16 ng/mL of TNF-α resulted in a less prominent effect on organoid morphology [21]; thus, we chose 16 ng/mL as a starting point. Similar to the increasing amounts of RAW 264.7 cells, higher concentrations of TNF-α had a more rapid and stronger effect on the organoids (Figure 2F). Specifically, with lower doses of TNF-α, some organoids retained their crypt-villus morphology and did not become spherical, whereas with higher doses, all of them became spherical after 5 days. However, when compared to the direct co-culture of organoids with macrophages, the effect of the added TNF-α on organoid morphology was less pronounced.

To test the functionality of the TNF-α-treated organoids and the organoids co-cultured with RAW 264.7 cells, we performed a proof-of-concept Forskolin assay. This is a standard assay for the quantification of the Cystic Fibrosis Transmembrane Conductance Regulator (CFTR) function [27]. It has been reported that TNF-α affects the CFTR function by stimulating CFTR-mediated fluid secretion [28]. We identified differences in the swelling of organoids among organoids cultured alone, organoids treated with TNF-α, and organoids co-cultured with RAW 264.7 macrophages (Supplementary Figure S2). Interestingly, in the last case, organoids did not seem to swell upon treatment with Forskolin. Future studies are required to shed more light on the mechanisms underlying these differences. The use of microwells can facilitate the performance of such tests in a high-throughput manner.

### 2.3. TNF-α-Induces Changes in Organoid Morphology

**Figure 3:**
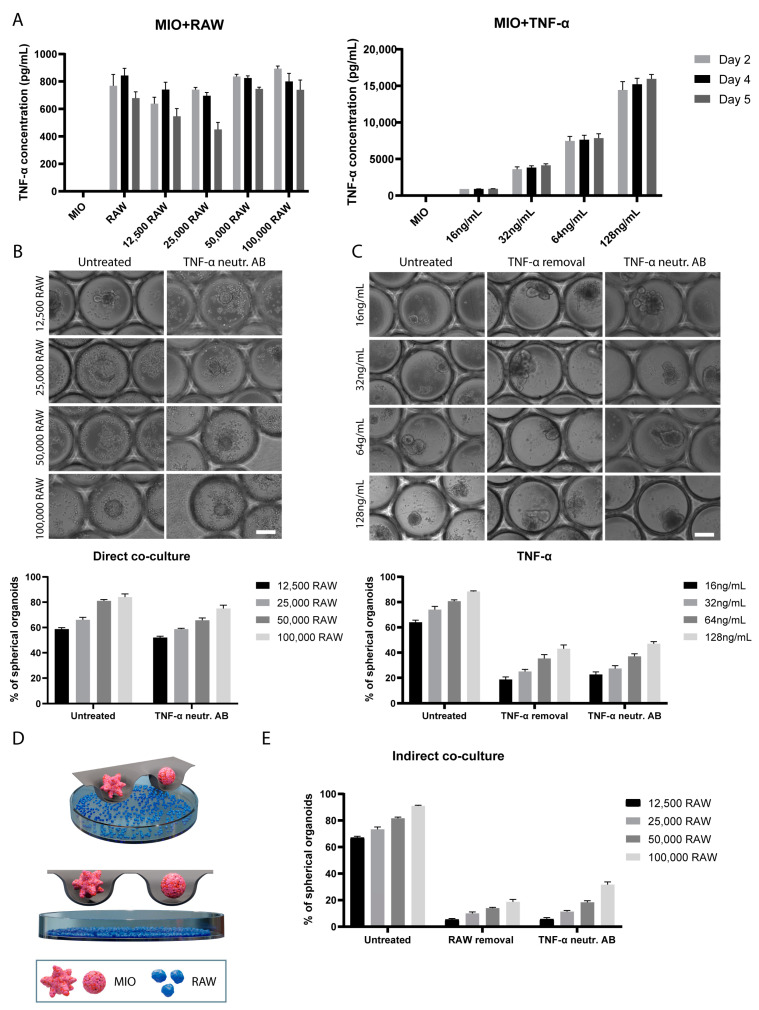
TNF-α-induced effects on organoid morphology. (**A**) Quantification of TNF-α concentration in the culture medium collected from organoids co-cultured with macrophages (left) and TNF-α-treated organoids (right) over a period of 5 days. Data are presented as the mean  ±  SEM. (*n* = 3). (**B**) Bright-field images demonstrating the morphology of organoids co-cultured with macrophages upon treatment with TNF-α neutralizing antibody (500 ng/mL). Graph indicates the percentages of spherical organoids with and without treatment. Data are presented as the mean  ±  SEM (*n* = 3). Scale bar represents 200 μm. (**C**) Bright-field images demonstrating the morphology of TNF-α-treated organoids upon additional treatment with TNF-α neutralizing antibody (500 ng/mL) and upon removal of TNF-α on day 3. Graph indicates the percentages of spherical organoids with and without additional treatments. Data are presented as the mean  ±  SEM (*n* = 3). Scale bar represents 200 μm. (**D**) Schematic illustration of the indirect co-culture of intestinal organoids with RAW 264.7 cells in microwell arrays. (**E**) Graph indicates the percentages of spherical organoids upon indirect co-culture with macrophages, indirect co-culture with subsequent removal of macrophages, and finally upon treatment with TNF-α neutralizing antibody on day 3 (500 ng/mL). Data are presented as the mean  ±  SEM (*n* = 3). Abbreviations: MIO, mouse intestinal organoids; RAW, RAW 264.7 cells.

To explore further the role of TNF-α on the morphological change in the organoids, we tested the removal of TNF-α from the culture and the addition of a TNF-α neutralizing antibody. We hypothesized that if the changes in the organoid morphology were solely dependent on TNF-α, neutralizing or completely removing TNF-α from the culture would allow organoids to rescue their crypt-villus morphology. Prior to testing this hypothesis, we aimed to determine the amount of TNF-α concentration in the organoid medium upon the co-culture of organoids with RAW 264.7 macrophages and the addition of TNF-α (Figure 3A). Similar concentrations of TNF-α were identified between RAW 264.7 cells cultured alone or in combination with organoids. Additionally, increasing numbers of RAW 264.7 cells increased the amounts of secreted TNF-α but not to a great extent. However, this was not the case for the added TNF-α, where higher amounts of added TNF-α resulted in a substantially higher protein concentration. It is worth noting that the TNF-α concentration was significantly higher when TNF-α was added to the medium (~0.9–15.96 ng/mL) compared to when organoids were co-cultured with RAW 264.7 cells (~0.45–0.8 ng/mL). Collectively, these results provided an indication about the amounts of TNF-α present in the culture medium.

Next, we tested the effects of a TNF-α neutralizing antibody and the removal of TNF-α from the medium by exchanging it with fresh medium (without TNF-α) on the organoids morphology. According to a previous study, when TNF-α neutralizing antibody was added to the culture medium, the TNF-α-induced morphological changes could be reversed [21]. Thus, we aimed to evaluate whether this occurs only when TNF-α is added to our microwell-based organoid culture or also when organoids are co-cultured with macrophages. When organoids are in direct contact with RAW 264.7 cells, the addition of TNF-α neutralizing antibody (500 ng/mL) on day 3 had a minimal effect, with less than 10% of organoids reversing their morphology from spherical to budding (Figure 3B). This indicates that even though the amount of neutralizing antibody added was enough to neutralize the effects of TNF-α (according to our ELISA measurements), the spherical morphology was retained. In contrast, the addition of neutralizing antibody or complete removal of TNF-α from the organoids (on day 3), which were initially treated with TNF-α, had a much more prominent effect on organoid morphology. We found that about 50% of the organoids that had initially become spherical returned to their crypt-villus architecture after treatment (Figure 3C). Collectively, these results indicate that, apart from TNF-α, other secreted factors and even the direct physical contact are involved in the morphological changes of the organoids, when they are in close proximity with RAW 264.7 cells.

### 2.4. Indirect Co-Culture of Intestinal Organoids and RAW 264.7 Macrophages

To investigate the interactions between the macrophages and the organoids and test whether the immediate physical contact between them is playing a role in the morphological changes, we performed the indirect co-culture of organoids with RAW 264.7 cells. Specifically, macrophages were seeded at the bottom of a 24-well plate and organoids were seeded inside the microwells (Figure 3D). Medium access was granted for both cell types both via the pores of the membrane and the sides of the insert. In this way, the crosstalk between the two cell types was mediated via the secretion of soluble factors by each cell line (paracrine signaling). This system is referred to as ‘indirect co-culture’. After 5 days of indirect co-culture, the vast majority of organoids had a spherical morphology. Specifically, when 12,500 RAW 264.7 cells were added, 61% of the organoids became spherical, whereas when 100,000 RAW 264.7 cells were added, 91% of the organoids became spherical. Hence, increasing numbers of RAW 264.7 cells resulted in increasing numbers of spherical organoids (Figure 3E).

Next, we assessed the morphology of the organoids after removing the RAW 264.7 cells from the culture or after adding TNF-α neutralizing antibody (500 ng/mL). Both treatments were found to have a similar effect on the organoid morphology. Almost all the organoids reversed their morphology and demonstrated budding architecture after 3 days (Figure 3E). Specifically, upon RAW 264.7 cells removal and TNF-α neutralizing antibody addition, 95% of the organoids co-cultured with 12,500 RAW 264.7 cells obtained a crypt-villus morphology. When co-cultured with 100,000 RAW 264.7 cells, 82% of the organoids obtained a crypt-villous morphology upon RAW 264.7 cells removal and 70% upon addition of TNF-α neutralizing antibody. Hence, TNF-α neutralizing antibody and RAW 264.7 cells removal have pronounced effects on organoid morphology when in indirect contact with macrophages, while, as discussed in the previous section, the effect of the TNF-α neutralizing antibody on organoids and macrophages in direct contact is less pronounced. Overall, these results indicate that there are prominent differences in the TNF-α-mediated morphological changes between organoids that are in direct or in indirect contact with macrophages.

### 2.5. Luminex Assay for TNF-α-Treated Intestinal Organoids

**Figure 4:**
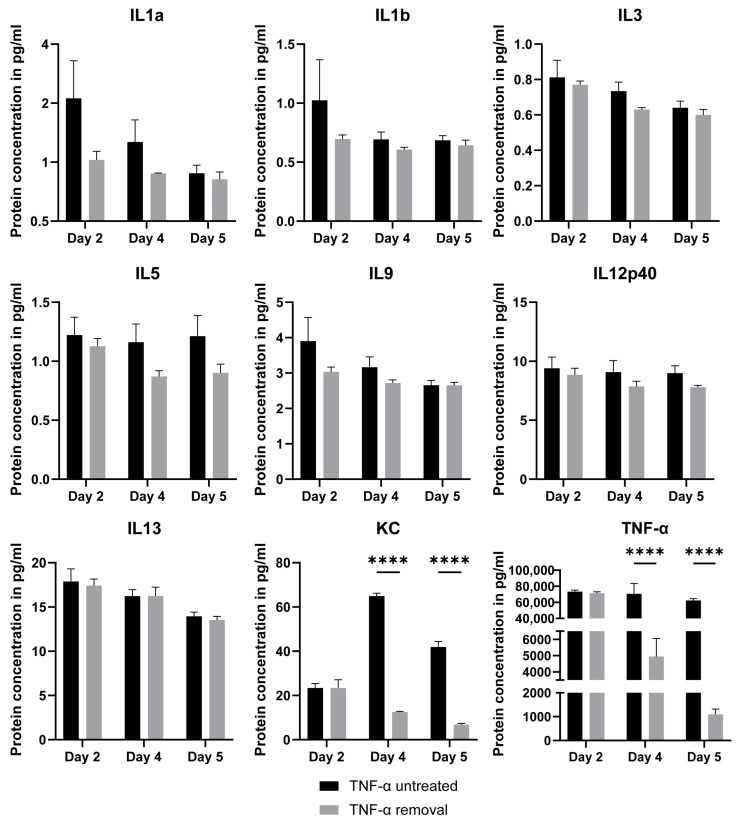
Luminex assay for TNF-α-treated intestinal organoids. Secretion of the cytokines IL1α, IL1β, IL3, IL5 IL9, IL12p40, IL13, KC, and TNF-α was identified in organoid culture medium upon addition of 64 ng/mL of TNF-α (TNF-α-untreated). Except for the KC and TNF-α, removal of TNF-α from the medium did not seem to strongly affect the production of these proteins. Data are presented as the mean  ±  SEM (*n* = 4). ****, for *p* < 0.0001.

To investigate the inflammatory responses of organoids upon exposure to the pro-inflammatory cytokine TNF-α and the mechanisms underlying the differences between the two co-culture systems and the TNF-α treatment further, we performed a Luminex assay. Specifically, we collected the cell culture medium upon stimulation of organoids with 64 ng/mL of TNF-α after 2, 4, and 5 days. We also tested the cytokine expression in organoids that were treated with TNF-α for 3 days, after which TNF-α was removed from the culture medium. We identified the expression of both pro- and anti-inflammatory cytokines that mediate immune responses. In particular, these included the interleukins IL1α, IL1β, IL3, IL5 IL9, IL12p40, IL13, keratinocyte chemoattractant (KC), and TNF-α (Figure 4). Interestingly, the secretion of these cytokines continued in similar levels even after the removal of TNF-α from the culture. Except for KC and TNF-α, there was no statistically significant difference in the cytokine secretion after TNF-α removal. These results indicate that intestinal organoids show an innate immune response function that is activated upon exposure to TNF-α. Furthermore, the treatment of organoids with TNF-α can induce the secretion of some of the cytokines in vitro, in a similar way as has been described for IBD situations [29].

### 2.6. Luminex Assay for Direct and Indirect Co-Culture of Intestinal Organoids with Macrophages

**Figure 5:**
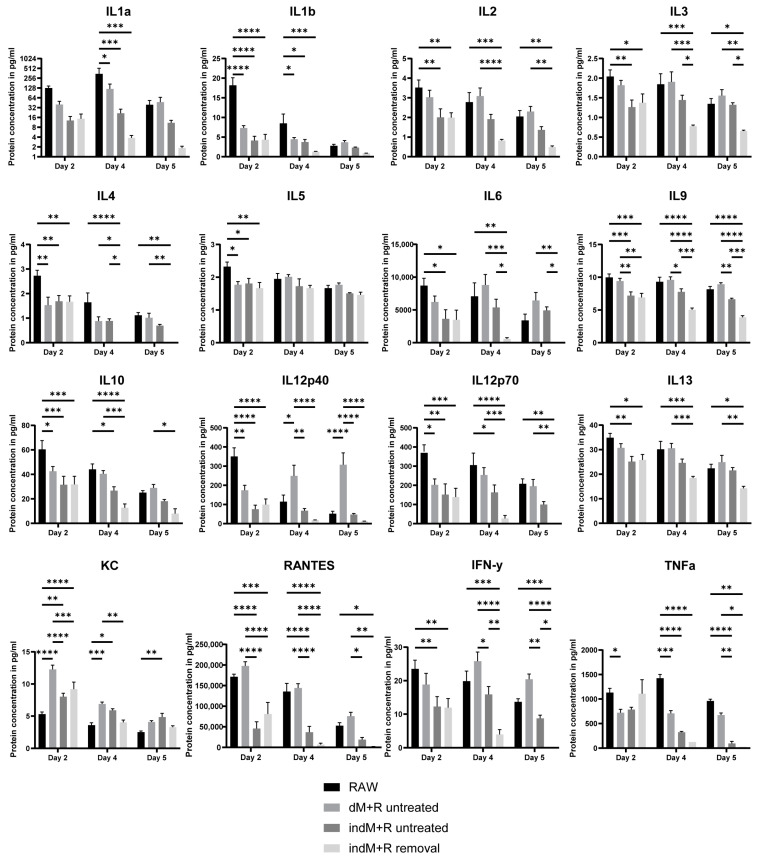
Luminex assay for direct and indirect co-culture of intestinal organoids with macrophages. Pro- and anti-inflammatory cytokines were produced upon direct and indirect co-culture of organoids with RAW 264.7 cells over a period of 5 days. Removal of RAW 264.7 cells was performed after 3 days of co-culture. Data are presented as the mean  ±  SEM (*n* = 4). ****, ***, **, and * for *p* < 0.0001, *p* < 0.001, *p* < 0.01, and *p* < 0.05, respectively. Abbreviations: RAW, RAW 264.7 cells; dM + R, direct co-culture; indM + R, indirect co-culture.

Next, we explored the cytokine release upon direct and indirect co-culture of intestinal organoids with 50,000 RAW 264.7 cells, after 2, 4, and 5 days. We also tested the secretion after removal of macrophages on day 3 from the culture (indirect culture only) and after monoculture of RAW 264.7 cells (50,000) in microwells. We identified a list of pro- and anti-inflammatory cytokines secreted, including IL1α, IL1β, IL2, IL3, IL4, IL5, IL6, IL9, IL10, IL12p40, IL12p70, IL13, KC, RANTES, Interferon (IFN)-γ, and TNF-α (Figure 5).

Interestingly, the secretion of most of these cytokines (except KC and RANTES) was higher when RAW 264.7 were cultured alone. This suggests a sort of immunomodulatory function of the intestinal organoids, which seem to be able to modulate the immune response of the macrophages. In addition, the fact that these proteins are produced even after the removal of RAW 264.7 cells further supports our previous suggestion that organoids demonstrate an innate immune response function upon exposure to inflammatory cytokines (added or secreted by the macrophages). Furthermore, in most cases, the secretion of these proteins seemed to be downregulated over time, when macrophages were cultured alone or co-cultured indirectly with organoids. In contrast, when organoids are in direct contact with the RAW 264.7 cells, the secretion is more stable over the 5 days of culture. It should be noted that the secreted levels of these proteins were higher when organoids were in direct contact with the macrophages compared to indirect co-culture. For most cytokines, these differences were statistically significant at all time-points. Hence, the close proximity of intestinal organoids with the macrophages seemed to have a prominent effect on the crosstalk between the cells.

## 3. Discussion

In the past few years, significant progress has been made in the development of intestinal organoid models. Multiple features of the in vivo intestine, such as the crypt-villus organization and functions such as barrier formation, drug absorption, and metabolism, can now be closely recapitulated by in vitro models. However, organoids still do not reflect the full complexity of the in vivo tissue, as important cues from surrounding tissues and cellular compartments are absent. Especially in the case of the intestine, the crosstalk between the epithelium and immune system is crucial to maintain homeostasis, as the intestine is constantly in contact with foreign materials. The development of more advanced 3D in vitro models incorporating immune components would be beneficial to unravel mechanisms involved in the defense system of the intestine but also for studying the pathogenesis of intestinal diseases, such as IBD. In this study, we incorporated immune cells in a microwell-based organoid model or added a pro-inflammatory cytokine and showed that organoids have an innate immunomodulatory function. This platform can be used to study intestinal inflammation in a more holistic way, as it allows modeling of both the intestinal epithelium and molecular/cellular components of the immune system and a controlled interaction between both compartments. Furthermore, this is a novel cell culture tool incorporating porous microwells for organoid culture in a compartmentalized, Transwell-like setup. In this system, organoids can be co-cultured with different cell types, which can grow either on the inner or the outer surfaces of the microwells, or on the bottom of the culture plate. In this way, the interactions of multiple factors can be studied within the same system. In future studies, different types of stromal cells (macrophages, T cells, dendritic cells, vascular cells, etc.) can be placed in several different configurations to decouple and explore the interactions between the compartments. Additionally, in our system organoids preserve their 3D architecture, whereas in traditional flat Transwell devices, organoids need to be dissociated to form cell monolayers [30,31,32,33,34]. This process requires a large number of cells and, hence a large number of organoids, which can be a pricey and time-consuming process. Thus, our microwell-based co-culture system can be particularly useful to study the interactions of the intestinal epithelium with immune and/or stromal cells.

Previously, efforts have been made to culture intestinal organoids in combination with immune cells. Specifically, mouse intestinal organoids have been co-cultured with RAW 264.7 macrophages before but, in this case, organoids were embedded in Matrigel and macrophages were cultured on a Transwell insert; thus, the crosstalk between the cells was not entirely representative of the in vivo situation [21]. In another study, human-monocyte-derived macrophages were co-cultured with human intestinal organoid-derived cell monolayers on opposite sides of a porous film [31]. Even though this model allows access to both apical and basolateral sides of the epithelium, the 3D architecture of the organoids is lost. Intraepithelial lymphocytes [35] and monocytes [36] were co-cultured with intestinal organoids inside Matrigel drops. However, these cell types are usually not embedded in Matrigel, which we hypothesize can affect their behavior as, in physiological circumstances, they are in suspension. Additionally, when embedded in Matrigel, organoids are not easy to track during culture. In contrast, when cultured in microwell arrays, organoids can be monitored easily throughout the culture period and can straightforwardly be harvested for further downstream analysis. Furthermore, in our system, the macrophages are added in suspension and then attached to the polymer films of the microwell arrays, thus behaving in a similar way to regular cell culture flasks. The close proximity of the organoids and the macrophages allowed for direct, physical interaction between the different cells in a more natural way. Apart from the small distance between the cells, the released cytokines did not have to diffuse over longer distances through Matrigel, which could also diminish and/or delay their effect. Overall, using a microwell system to perform co-culture allows for controlled positioning of the different cell types, which can benefit the modeling of certain in vivo conditions, and allows for a controlled decoupling of specific factors/interactions. For example, in IBD, there is infiltration of macrophages in the intestinal mucosa, which can only be achieved *in vitro* if cells are placed nearby and the cells can actively migrate.

The difference between the direct and indirect contact of organoids with macrophages was shown here both by the differences in the morphology of the organoids, but also in the series of released cytokines. The organoids’ morphology was changed from crypt-villus to spherical upon co-culture with macrophages, and one of the key mechanisms underlying this change is the release of the pro-inflammatory cytokine TNF-α. TNF-α has been associated with the loss of epithelial barrier integrity, via the phosphorylation of myosin light chain, the loss of zonula occludens-1, and the internalization of occludin within the cytoplasm [37,38,39]. Treatment with TNF-α neutralizing antibody failed to rescue the organoid architecture from spherical to budding when organoids were in direct contact with macrophages, whereas this was not the case when they were in indirect contact. In addition, the secretion of cytokines was higher and more stable over time when organoids were in direct contact with the macrophages, indicating that receptor interactions may play a pivotal role. Thus, the direct co-culture system resembles more the conditions found in chronic intestinal inflammation, where we have excessive secretion of cytokines. The indirect system seems to better reflect conditions of a mild acute inflammation, where the secretion of cytokines remains lower and the immune response is less excessive. This notion is also supported by the fact that upon removal of RAW 264.7 cells following indirect co-culture with organoids, the effects of the secreted cytokines are minimized or even nullified.

In a comparison of immune responses between the co-culture of organoids with macrophages and treatment with TNF-α, we identified that IL1α, IL1β, IL3, IL5, IL9, IL12p40, IL13, KC, and TNF-α were secreted in both conditions, whereas IL2, IL4, IL6, IL10, IL12p70, RANTES, and IFN-γ were solely secreted in the co-culture. The roles of all these proteins have been associated previously with acute and/or chronic intestinal inflammatory responses [40]. For example, the pro-inflammatory cytokines IL1α, IL1β, IL2, IL6, IL12, IFN-γ, and TNF-α have been linked with initiation and progression of IBD, whereas the anti-inflammatory IL4, IL10, and IL13 have been associated with the pathogenesis of IBD by decreasing the production of pro-inflammatory cytokines [29,41,42]. Similarly, IL3 and IL5 have been regarded as pleiotropic regulators of inflammation and they are implicated in reducing intestinal inflammation [43,44]. IL9 has also been found to play a role in the pathogenesis of IBD and has been suggested as a disease severity marker and potential therapeutic candidate [45]. RANTES is a chemokine that is upregulated in IBD and the identification of differences in its expression patterns (granulomatous vs. non-granulomatous) has been proposed as a possible method to distinguish between Crohn’s disease and ulcerative colitis [46,47]. RANTES is also upregulated in gastrointestinal tumors [48]. Finally, KC is a chemokine that is induced by IL1 and TNF-α, and its expression is upregulated in IBD [49,50]. Collectively, these data indicate that a co-culture system of intestinal organoids with macrophages provides a more holistic approach to study inflammatory responses than an exogenous treatment of organoids with TNF-α only. However, this is still a simplified model compared to the in vivo situation, where more immune cell types are involved (e.g., T-cells and innate lymphoid cells) and more cytokines (e.g., IL8, IL17, and IL21) are present during inflammatory responses. In the future, it would be interesting to use human cells and explore whether similar responses can be reproduced. It would also be intriguing to test different types or even combinations of immune cells and assess the directionality of the interactions among them. Using patient-derived organoids, treatments against more cytokines than just TNF-α could be explored, aiming for more efficient and even personalized therapies.

To conclude, we developed a versatile microwell-based platform to perform co-culture of intestinal organoids with RAW 264.7 macrophages. Within this system, the positioning of the different cells can be controlled and the interactions of multiple factors can be studied simultaneously. Here, we placed RAW 264.7 cells either in direct contact with the intestinal organoids or in indirect contact, and in both systems, we identified characteristic secretion profiles of cytokines involved in inflammatory responses. These responses resembled aspects of the early and later phases (acute and more chronic) of inflammation. This indicates that our novel systems can be used to gain new insights into the mechanisms and interactions underlying intestinal inflammation. The microwell-based culture system facilitates a continuous monitoring of the macrophage–organoid interactions and the performance of high-throughput assays. For example, these models can be valuable alternatives for the development of more effective drug treatments against intestinal inflammatory diseases.

## 4. Materials and Methods

### 4.1. Fabrication and Preparation of Microwells for Organoid Culture

Microwell arrays were fabricated using microthermoforming as previously described [17,51]. Briefly, 50 μm thin polycarbonate films were used to create arrays of 289 microwells. Morphometric characterization was performed using a confocal laser scanning microscope (Keyence 3D Laser Scanning Microscope VK-X250K; Keyence, Mechelen, Belgium) and the corresponding analysis software (Keyence MultiFileAnalyzer; v1.3.1.120). Each microwell had a diameter of 500 μm and a depth of ~300 μm. For the indirect co-culture experiments, porous membranes with the same dimensions were used. The pore size was 0.8 μm and the pore density was 1E06. Prior to cell culture, microwell arrays were punched to the size of a well of a 24-well plate and pre-wetted and sterilized in a graded series of 2-isopropanol (100%, 70%, 50%, 25%, and 10%; VWR). Subsequently, they were washed twice in Dulbecco’s phosphate-buffered saline (PBS; Sigma-Aldrich, Darmstadt, Germany) and placed at the bottom of non-tissue-culture-treated 24-well plates, where they were kept in place by elastomeric O-rings (ERIKS).

### 4.2. Intestinal Organoid Culture

Mouse intestinal organoids derived from the small intestine of C57BL/6 mice were purchased as cryopreserved fragments (STEMCELL Technologies, Vancouver, BC, Canada). Organoids were cultured and embedded in Matrigel domes, as previously described [9] with minor modifications. Briefly, a 50:50 mixture of IntestiCult Organoid Growth Medium (STEMCELL Technologies) and Matrigel (Corning, New York, NY, USA) containing organoids was placed dropwise in tissue-culture-treated 24-well plates. After polymerization at 37 °C and 5% CO_2_ for 10 min, Matrigel domes were covered with 650 μL of IntestiCult. Medium was refreshed every 2 days. Passaging of the organoids was performed every 5–7 days using Gentle Cell Dissociation Reagent (GCDR; STEMCELL Technologies).

To seed the organoid fragments in the microwells, following the organoid dissociation process, fragments were resuspended in IntestiCult medium supplemented with 2% Matrigel. Then, 50 μL of this mixture was added onto the microwell arrays, and fragments were let to settle for 2 h by gravity, before adding more medium.

### 4.3. Macrophage Culture

The murine macrophage cell line RAW 264.7 was purchased from ECACC. Cells were cultured in Dulbecco’s modified Eagle’s medium (DMEM; Gibco, New York, NY, USA) supplemented with 10% fetal bovine serum (FBS; Sigma-Aldrich) and passaged every 3–4 days upon reaching confluence. The maximum passage number of the cells used in this study was 15.

### 4.4. Co-Culture of Intestinal Organoids and Macrophages

To perform direct co-culture, initially, RAW 264.7 cells were seeded into the microwells. After centrifugation to accelerate the inoculation of the cells into the microwells, organoid fragments were added to the culture. The system was placed at 37 °C and 5% CO_2_ for 2 h, so that the organoids settle into the microwells as well, and afterward, more organoid medium was added. Different concentrations of RAW 264.7 cells were tested, ranging from 12,500 cells/well to 100,000 cells/well.

To perform indirect co-culture, RAW 264.7 cells were initially seeded at the bottom of the tissue-culture-treated 24-well plate. Similar to the direct co-culture, different concentrations of RAW 264.7 cells were tested (12,500, 25,000, 50,000, and 100,000). Following that, a porous microwell array was placed in the same well, and organoid fragments were seeded into the microwells. To avoid contact of the microwell array with the macrophages, an O-ring was placed underneath the microwell array.

### 4.5. Immunofluorescence and Confocal Microscopy

Initially, organoids and cells were fixed with 4% paraformaldehyde (VWR, Amsterdam, The Netherlands) in PBS. Next, permeabilization was performed with 0.5% Triton X-100 (Merck, Rahway, NJ, USA) in PBS for 30 min at room temperature (RT). For blocking, cells/organoids were incubated with 5% donkey serum (VWR) in permeabilization solution for 30 min at RT. Afterward, primary antibodies against Lysozyme (Lyz1; Agilent, Santa Clara, CA, USA), Ki67 (Abcam, Cambridge, UK), CD11b (Abcam), and F4/80 (Abcam) diluted in blocking buffer were added and incubated overnight at 4 °C. The following day, the secondary antibodies and, when applicable, phalloidin (Thermofisher, Waltham, MA, USA) were incubated for 2 h at RT. Finally, samples were counterstained with 4′,6-diamidino-2-phenylindole (DAPI; Sigma-Aldrich) and mounted using Dako Fluorescence Mounting Medium (Agilent). All stained samples were imaged using a TCS SP8 confocal laser scanning microscope (Leica, Wetzlar, Germany) and processed with ImageJ.

### 4.6. Enzyme-Linked Immunosorbent Assay (ELISA)

The intestinal organoid medium was collected following the direct co-culture of organoids with RAW 264.7 cells (12,500, 25,000, 50,000, and 100,000 cells) or the addition of TNF-α (16, 32, 64, and 128 ng/mL) over three time points (days 2, 4, and 5). The amount of TNF-α protein was measured with ELISA (R&D Systems, Minneapolis, MN, USA) according to the manufacturer’s instructions. Absorbance was measured at 450 nm using a CLARIOstar plate reader (BMG LABTECH, Ortenberg, Germany).

### 4.7. Luminex Assay

The intestinal organoid medium was collected following the direct and indirect co-culture of organoids with RAW 264.7 cells or the addition of TNF-α and subsequent treatments (removal of TNF-α and/or removal of RAW 264.7 cells) over three time-points (days 2, 4, and 5). The amounts of cytokines were detected by the Bio-Plex Pro Mouse Cytokine 23-plex Assay, according to the manufacturer’s guidelines. Fluorescence intensity was measured using a Luminex 100 Bio-Plex Liquid Array Multiplexing System (Bio-Rad, Hercules, CA, USA).

### 4.8. Scanning Electron Microscopy (SEM)

Microwell arrays were mounted on SEM stubs, sputter-coated with a thin layer of gold using a SC7620 Mini Sputter Coater (Quorum Technologies, London, UK), and finally examined using a JSM-IT200 electron microscope (Jeol, Nieuw-Vennep, Benelux)).

### 4.9. Forskolin Assay

Intestinal organoids cultured alone, treated with TNF-α, and co-cultured with RAW 264.7 macrophages were treated with 5 μM Forskolin and directly analyzed by live cell microscopy (Nikon Inverted Research Microscope ECLIPSE Ti, Amstelveen, The Netherlands).

### 4.10. Statistical Analysis

Statistical analysis was performed using Prism 9 software (GraphPad). Two-way ANOVA followed by Tukey’s test were used to determine statistical significance. Significant differences were defined as *p* < 0.05. *p* values of statistical significance are represented as ****, ***, **, and * for *p* < 0.0001, *p* < 0.001, *p* < 0.01, and *p* < 0.05, respectively.

## Data Availability

Data available on request from the corresponding author.

## Supplementary Materials

The following supporting information can be downloaded at: https://www.mdpi.com/article/10.3390/ijms232315364/s1.
