# Supplementary material for: A Microwell-Based Intestinal Organoid-Macrophage Co-Culture System to Study Intestinal Inflammation
Source: Int J Mol Sci. 2022 Dec 6;23(23):15364. doi: 10.3390/ijms232315364 (PMC9736416; doi:10.3390/ijms232315364)
Supplement: Supplementary file 1 [file ijms-23-15364-s001.zip › ijms-2020294-supplementary/ijms-2020294-supplementary.pdf]

Supplementary Material

A Microwell-Based Intestinal Organoid-Macrophage Co-Culture System to Study Intestinal Inflammation

Panagiota Kakni [1], Roman Truckenmüller [1], Pamela Habibović [1], Martijn van Griensven [2] and Stefan Giselbrecht [1,*]

Supplementary table

Table S1. Description of the culture conditions used for the experiments

**Name**	**Culture Conditions**
MIO	Mouse intestinal organoids cultured in microwells
RAW	RAW cells cultured in microwells
TNF-α untreated	MIO treated with TNF-α for 5 days
TNF-α neutralizing antibody	MIO treated with TNF-α for 5 days and TNF-α neutralizing antibody from day 3 onwards
TNF-α removal	MIO treated with TNF-α until day 3 and then remove TNF-α from the culture medium
dMIO+RAW untreated	Direct co-culture of MIO with RAW for 5 days
indMIO+RAW untreated	Indirect co-culture of MIO with RAW for 5 days
indMIO+RAW removal	Indirect co-culture of MIO with RAW and on day 3 removal of RAW cells

Supplementary figures

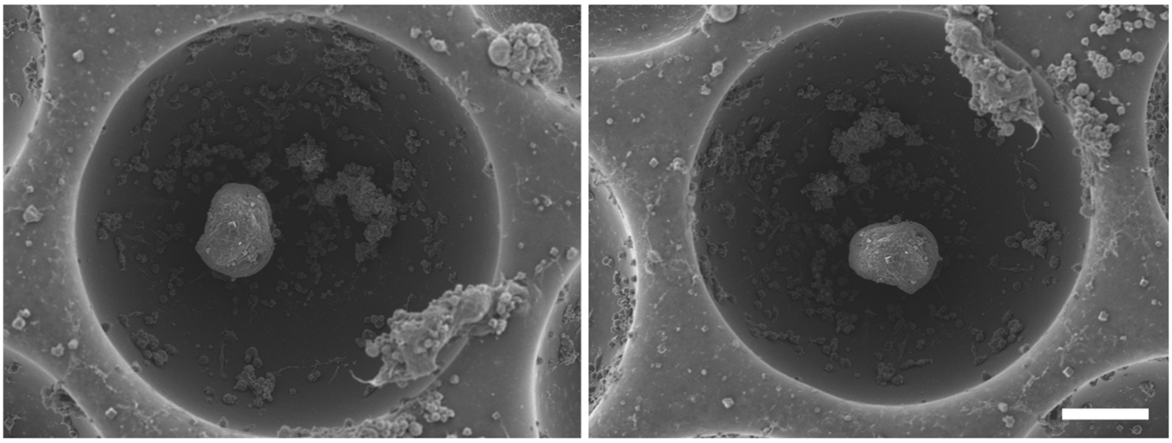

Figure S1. SEM images demonstrating the direct co-culture of intestinal organoids with RAW 264.7 cells. Scale bar represents 100 μm.

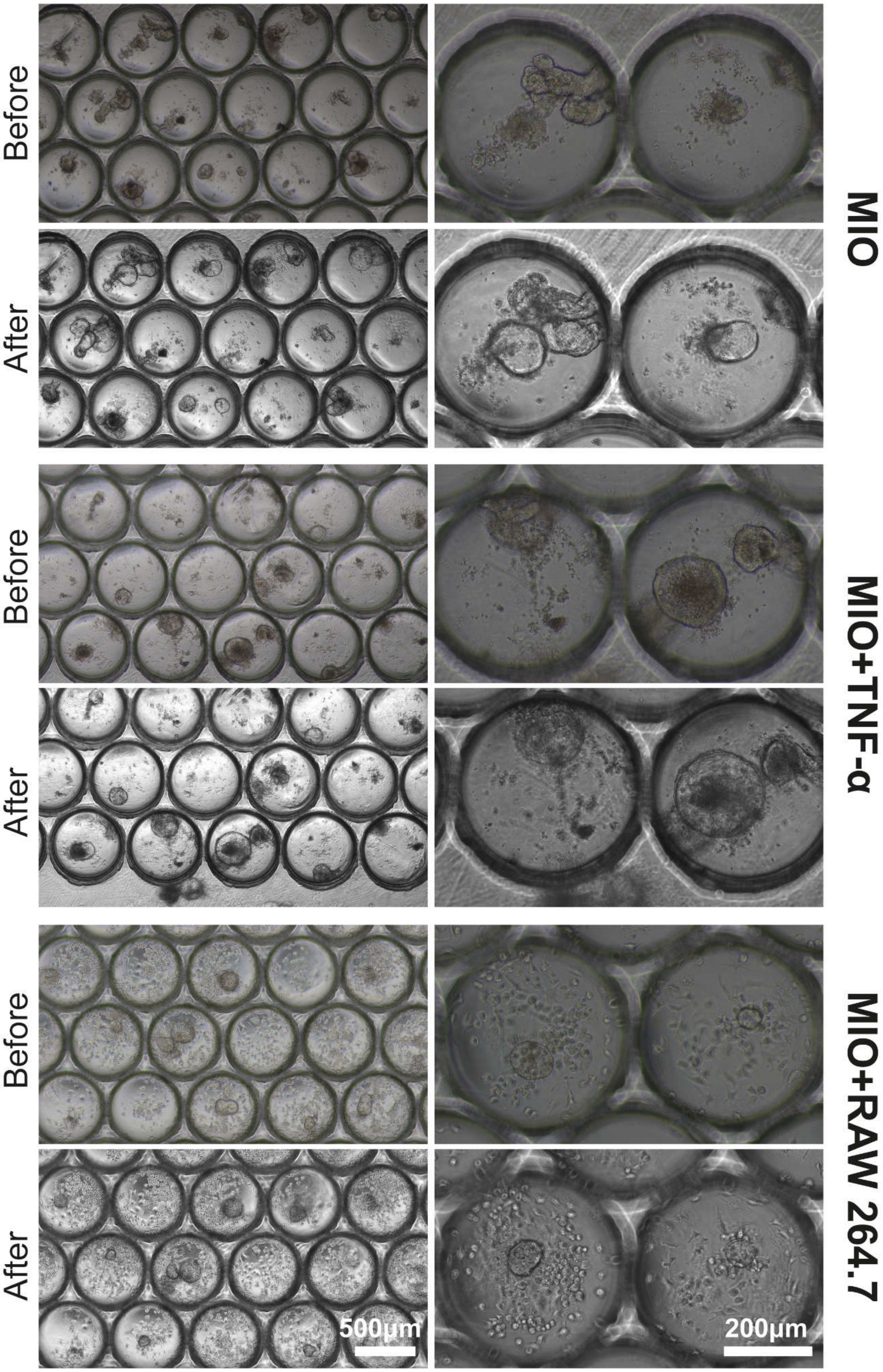

Figure S2. Bright-field images demonstrating the swelling of organoids upon treatment with 5 μM Forskolin for 3 hours.

Supplementary video

Video S1. Time-lapse imaging of the direct co-culture of intestinal organoids with 50,000 RAW 264.7 cells. Single plane images were taken every 1 h for 3 days.
